# Acute exposure to high‐induction electromagnetic field affects activity of model peripheral sensory neurons

**DOI:** 10.1111/jcmm.13423

**Published:** 2017-12-06

**Authors:** Jaroslav Prucha, Jan Krusek, Ivan Dittert, Viktor Sinica, Anna Kadkova, Viktorie Vlachova

**Affiliations:** ^1^ Department of Information and Communication Technologies in Medicine Czech Technical University in Prague Prague Czech Republic; ^2^ Department of Health Care Disciplines and Population Protection Faculty of Biomedical Engineering Czech Technical University in Prague Prague Czech Republic; ^3^ Department of Cellular Neurophysiology Institute of Physiology, Czech Academy of Sciences Prague Czech Republic

**Keywords:** electromagnetic field, primary sensory neuron, ion channel, bradykinin receptor, transient receptor potential channel

## Abstract

Exposure to repetitive low‐frequency electromagnetic field (LF‐EMF) shows promise as a non‐invasive approach to treat various sensory and neurological disorders. Despite considerable progress in the development of modern stimulation devices, there is a limited understanding of the mechanisms underlying their biological effects and potential targets at the cellular level. A significant impact of electromagnetic field on voltage‐gated calcium channels and downstream signalling pathways has been convincingly demonstrated in many distinct cell types. However, evidence for clear effects on primary sensory neurons that particularly may be responsible for the analgesic actions of LF‐EMF is still lacking. Here, we used F11 cells derived from dorsal root ganglia neurons as an *in vitro* model of peripheral sensory neurons and three different protocols of high‐induction magnetic stimulation to determine the effects on chemical responsiveness and spontaneous activity. We show that short‐term (<180 sec.) exposure of F11 cells to LF‐EMF reduces calcium transients in response to bradykinin, a potent pain‐producing inflammatory agent formed at sites of injury. Moreover, we characterize an immediate and reversible potentiating effect of LF‐EMF on neuronal spontaneous activity. Our results provide new evidence that electromagnetic field may directly modulate the activity of sensory neurons and highlight the potential of sensory neuron‐derived cell line as a tool for studying the underlying mechanisms at the cellular and molecular level.

## Introduction

Diverse cellular and physiological effects induced by exposure to repetitive low‐frequency electromagnetic field (LF‐EMF, <300 Hz) have a common denominator: calcium homoeostasis. Calcium ions (Ca^2+^) control processes as critical as cell proliferation, differentiation, neuronal development, muscle contraction, transmitter release, vesicle endocytosis, gene transcription and cell death 1. All these events have been convincingly shown to be affected by LF‐EMF, and there is a large body of literature suggesting that activation of voltage‐gated Ca^2+^ channels and downstream intracellular responses produce most of the LF‐EMF effects (e.g. Ref. 2, 3, 4, 5, 6 and references therein). However, recent evidence suggests that LF‐EMF may also primarily act via up‐regulation of arachidonic acid and its metabolites, prostaglandin E2 or leukotriene E4. These inflammatory mediators sensitize excitatory (sodium) or inhibitory (γ‐aminobutyric acid type A) ion channels and reduce the activity of low‐voltage‐gated T‐type calcium channels 5, 7, 8. The differential mechanisms of modulation may underlie diverse effects of LF‐EMF which could have beneficial or deleterious outcome depending on the cell type and cell environment 2, 9. It is increasingly understood that cellular processes affected by exposure to high‐induction electromagnetic field have to be considered and appropriately characterized in a strictly cellular‐specific context to assure a rational and safe usage of electromagnetic stimulators in future clinical practice 10.

High‐induction electromagnetic field applied peripherally over a muscle or spinal nerve roots is capable of improving sensorimotor impairments and reducing acute and persistent pain 11, 12, 13, 14, 15. Proprioceptive as well as superficial cutaneous and nociceptive afferents may be recruited in these processes, although the latter are generally thought to be less involved 15. Recent advances in single cell transcriptomics enabling to dissect sensory responsive cells into distinct neuronal subtypes 16 together with the ever‐increasing information available on the multitude of specific receptors and channels involved in sensory transduction and nociception 17, 18 have opened an exciting opportunity to study the effects of LF‐EMF in a manner even more focused on cellular and molecular mechanisms. Particularly, the recent systematic functional and transcriptomic characterization of neuroblastoma cells derived from dorsal root ganglia neurons 19 demonstrated that F11 cell line represents a convenient model to study the cellular mechanisms of sensory transduction and transmission *in vitro*. These cells endogenously express a well‐characterized range of receptors and ion channels with potential roles in neuronal signalling and intracellular pathways implicated in various sensory functions, including those for nociceptive sensory neurons 19, 20, 21, 22: voltage‐gated K^+^ and Na^+^ channels (particularly Na_V_1.6, Na_V_1.7 and K_V_11.1), and many other ion channels and receptors with recognized roles in neuronal excitability. Moreover, F11 cells exhibit robust Ca^2+^ transients in response to subnanomolar concentrations of bradykinin 20, 22, a potent algogenic nonapeptide that is known to be released *in vivo* by tissue damage and under inflammatory conditions 23. These responses are mediated by bradykinin B2 and, to a lesser extent, B1 receptors that belong to a family of G‐protein coupled receptors and activate the G_q_/phospholipase C pathway, catalysing the hydrolysis of phosphatidylinositol 4,5‐bisphosphate and the production of the two second messengers, inositol triphosphate (IP_3_) and diacylglycerol (DAG). The former product of hydrolysis activates intracellular Ca^2+^ release via IP_3_ receptor channels located on the endoplasmic reticulum, whereas the membrane‐bound DAG activates protein kinase C (PKC). Upon tissue trauma, inflammation or nerve injury, the B1 receptors are up‐regulated and together with constitutive B2 receptors contribute to the development of pain and hyperalgesia not only in periphery but also in the spinal cord and higher centres 23.

The recent findings on F11 cells providing a well‐characterized cellular model of peripheral sensory neurons 19 and the widely accepted important role of bradykinin as a mediator of pain and inflammation 23 prompted us to investigate the impact of LF‐EMF on calcium responses induced in naïve and transfected F11 cells. We focus on acute changes because their understanding would greatly help our further comprehension of the molecular and cellular mechanisms that initiate the analgesic effects of LF‐EMF.

## Materials and methods

### LF‐EMF exposure systems

Three different systems for repetitive electromagnetic stimulation were used: EMF1, in physiotherapy so‐called electrodeless electrotherapy, type name VAS‐07 STRONG (EMBITRON s.r.o., Plzen, Czech Republic) generated, by means of the ring coil (7 cm middle radius), rectangular bipolar impulses of the induced electrical field of the intensity of 0.2–2 V/m and, consequently, electrical current density between 0.1 and 1 A/m^2^ at the average tissue conductivity of 0.5 S/m. The positive part of the impulses had time duration of 340 μs, and the negative part, with a smaller amplitude, had time duration approximately 4.5 ms (this is the “repetitive single pulse” according to C.A.L. Bassett 24, and Ref. see 25 and references therein). The repetitive frequency was 72 Hz. Carrier magnetic field had an average amplitude of 5 mT. EMF2, in physiotherapy so‐called high‐induction magnetic stimulation, medical device of type name “SALUTER MOTI” (EMBITRON s.r.o., Plzen, Czech Republic), was produced by an electromagnetic generator system characterized by magnetic induction of amplitude of about 2 T, sinusoidal bipolar pulses with a width of 350 μs. The induced electrical field of the intensity of 20–200 V/m and consequently, electrical current density was between 10 and 100 A/m^2^ at the average tissue conductivity of 0.5 S/m. The stimulator induced a repetitive 10 sec. packet of pulses at a frequency of 25 Hz or 1 Hz with respectively 50 sec. (at 25 Hz) or 2 sec. (at 1 Hz) after‐pause. EMF3, in physiotherapy so‐called high‐induction magnetic stimulation, medical device type name “DIPOL SETA‐D, I‐100” (NPF Dipol, Vitebsk, Belarus), generated 170 μs rectangular pulses of magnetic field, in the burst of six pulses, with a delay between the pulses about 500 ms, one burst takes approximately 2.5 sec., followed by a 3.5 sec. pause, hence, the frequency of the bursts was 10 per minute. Magnetic induction amplitude was about 1 T, the induced electrical field was roughly 20 V/m and consequently, electrical current density was 10 A/m^2^ at the average tissue conductivity of 0.5 S/m. In all cases, we took care to orient the system so that the surface of the culture dish bottom was as perpendicular as possible to the lines of force of the alternating magnetic field. Changes of temperature around the recording area were measured by a sensitive digital thermometer before and after each recording. If the temperature changed more than ~1°C, the data were discarded.

### Cell cultures and transfection

F11 cells (The European Collection of Authenticated Cell Cultures) cultured in Dulbecco's modified Eagle's medium (DMEM) supplemented with 2 mM glutamine and 10% foetal bovine serum were passaged once a week using trypsin‐EDTA (Invitrogen, Carlsbad, CA, USA) and grown under 5% CO_2_ at 37°C. For experiments, cells were grown for 7–11 days to properly differentiate. The day before transfection, cells were plated in 24‐well plates (2 × 10^5^ cells per well) in 0.5 ml of medium and became confluent on the day of transfection. The cells were transiently cotransfected with 300 ng of cDNA plasmid encoding human TRPA1 (in the pCMV6‐XL4 vector, OriGene Technologies, Rockville, MD, USA) and with 200 ng of GFP plasmid (TaKaRa, Japan) with the use of Lipofectamine 2000 (Invitrogen) and then plated on poly‐L‐lysine‐coated glass coverslips.

### Measurement of intracellular Ca^2+^ responses

The cells were loaded with 1 μM Fura‐2‐AM (Invitrogen) dissolved in a bath solution (160 mM NaCl, 2.5 mM KCl, 1 mM CaCl_2_, 2 mM MgCl_2_, 10 mM HEPES, 10 mM glucose, adjusted to pH 7.3 with NaOH, 320 mOsm) for 1 hr followed by a 20‐min. wash in fresh bath solution. The Cell^R imaging system (Olympus Biosystems, Planegg, Germany) was used to capture the fluorescence images obtained with alternating excitation at 340 and 380 nm (TILL Photonics, Planneg, Germany) and emission at ˃510 nm. The metal xy positioning microscope stage (Olympus IX81) was replaced by plastic stage to prevent EMF‐induced mechanical resonance that impaired the quality of image recording. Emission ratios were calculated for each 0.5‐sec. interval after subtraction of the background. Spontaneous activity of cells was measured using excitation/emission wavelengths 380 nm/510 nm and sampling rate ≤500 ms. A system for rapid superfusion of the cultured cells was used for drug application 26. The control experiments were performed for each electromagnetic exposure system during the same day for comparison.

### Electrophysiology

Whole‐cell membrane currents were recorded by employing an Axopatch 200B amplifier and pCLAMP 10 software (Molecular Devices), exactly as described previously 27. The extracellular bath solutions contained the following: 160 mM NaCl, 2.5 mM KCl, 1 mM CaCl_2_, 2 mM MgCl_2_, 10 mM HEPES, 10 mM glucose, adjusted to pH 7.3 and 320 mOsm. The internal pipette solution contained 125 mM K^+^‐gluconate, 15 mM KCl, 5 mM EGTA, 0.5 mM CaCl_2_, 10 mM HEPES and 2 mM MgATP, 0.3 mM NaGTP, 10 mM creatine phosphate adjusted with KOH to pH 7.2 and 300 mOsm. Only one recording was performed on any one coverslip of cells to ensure that recordings were made from cells not previously exposed to chemical stimuli.

### Statistical analysis

All of the data were analysed using ImageJ software (NIH, Bethesda, MD, USA), pCLAMP 10 (Molecular Devices) and SigmaPlot 10 (Systat Software Inc., San Jose, CA, USA). Statistical significance was determined by Student's *t‐*test; differences were considered significant at *P* < 0.05 where not stated otherwise. The data are presented as means ± S.E.M.

## Results

### LF‐EMF inhibits bradykinin‐induced calcium responses in F11 cells

**Figure 1:**
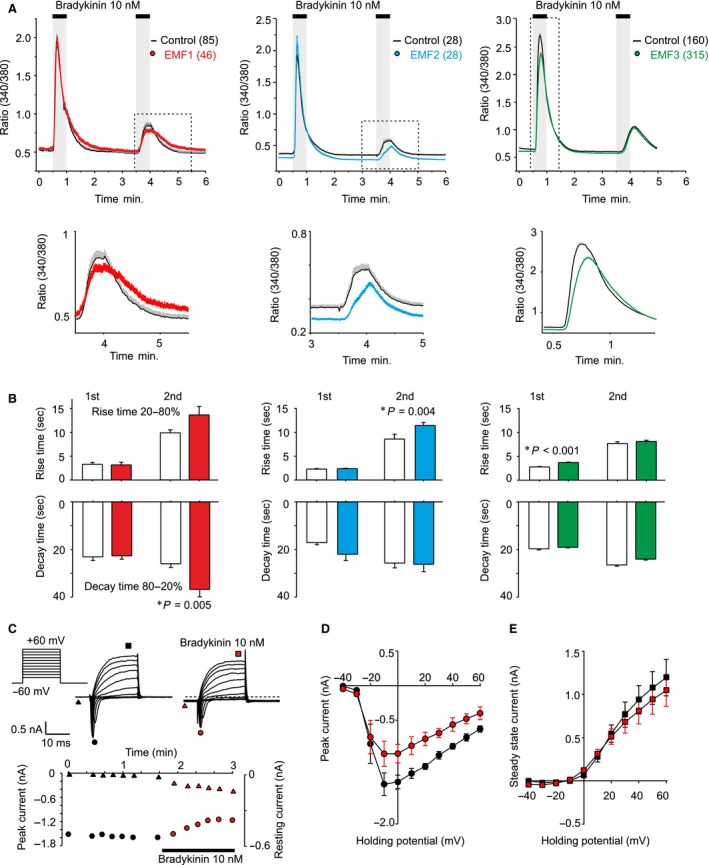
Exposure to high‐induction electromagnetic field attenuates responses to 10 nM bradykinin in cultured F11 cells. (**A**) Average calcium responses obtained from control cells and from cells continuously exposed to three different types of electromagnetic field (EMF1, EMF2, EMF3, see Materials and Methods). Continuous curves are the mean, colour envelopes the +S.E.M. (*n* indicated in parentheses). Magnified view of average data in dotted box is shown below each graph. The electromagnetic field was switched on 30 sec. before the recording. (**B**) Summary of the effects of LF‐EMF on average rise time (20–80%) and average decay time (80–20%) constants of the second (in case of EMF1 and EMF2) and the first Ca^2+^ response (in case of EMF3) to bradykinin. (**C**) Whole‐cell patch‐clamp recording from a typical naïve F11 cell showing Na^+^ and K^+^ responses to repeated stimulation by depolarizing steps from −40 mV to +60 mV (holding potential −60 mV) in the absence and in the presence of bradykinin. Graph below shows the effects of bradykinin on the inward peak current (left axis, circles) and the resting membrane current (right axis, triangles). Note that upon bradykinin application, the resting inward membrane current increased from −4 pA to −143 pA. Effects of bradykinin on the average current‐voltage relationship of the peak amplitude (**D**, indicated by circles in C), and the steady‐state current (**E**, indicated by squares in C). Data are represented as mean ± S.E.M. (*n* = 3).

We examined the effects of three distinct sources of repetitive LF‐EMF on endogenous Ca^2+^ responses of F11 cells to repeated applications of 10 nM bradykinin (BK). We applied two subsequent BK exposures, each lasting 30 sec. and separated by 3 min. In the control experiments (without EMF1 or EMF2), the first application of BK induced Ca^2+^ responses with peak amplitudes of 1.96 ± 0.11 and 1.93 ± 0.09 (*n* = 85 and 28; ratio 340/380 nm in arbitrary units). The second response was largely reduced (to 0.85 ± 0.04 and 0.57 ± 0.04; *P* = <0.001). As shown in Figure 1, continuous exposure to EMF1 or EMF2 did not significantly influence the average initial response to BK (1.94 ± 0.11 and 2.12 ± 0.15, *n* = 46 and 28; *P* = 0.907 and 0.287), but the subsequent response to BK applied three minutes later was markedly suppressed (*P* = 0.047 and 0.031) and delayed. Upon exposure to EMF1, the 80–20% decay time of the second response was significantly slowed from 26 ± 2 to 37 ± 3 sec. (*P* = 0.005). Exposure to EMF3 caused a significantly delayed onset and reduction of the first average response from 2.7 ± 0.1 to 2.3 ± 0.1 (*n* = 160 and 315; *P* = <0.001) (Fig. 1B). The 20–80% rise time of the first BK responses significantly increased from 2.3 ± 0.1 sec. to 3.7 ± 0.1 sec. (*P* < 0.001). The second average BK response upon exposure to EMF3 was no different from control cells. To gain qualitative information about the overall effects of bradykinin on F11 cell excitability, we used patch‐clamp technique and measured membrane currents in response to 20‐ms voltage pulses from −40 mV to +60 mV in the absence and in the presence of 10 nM bradykinin (Fig. 1C). The inward currents, which are typical for voltage‐gated Na^+^ channels, were significantly inhibited by bradykinin (from −1.39 ± 0.10 nA to −0.98 ± 0.11 nA; *n* = 3, *P* < 0.05) and the resting membrane current increased (Fig. 1C and D). The outward voltage‐dependent potassium currents were only slightly affected (Fig. 1E).

### F11 cells exhibit spontaneous activity, that is increased in response to LF‐EMF

**Figure 2:**
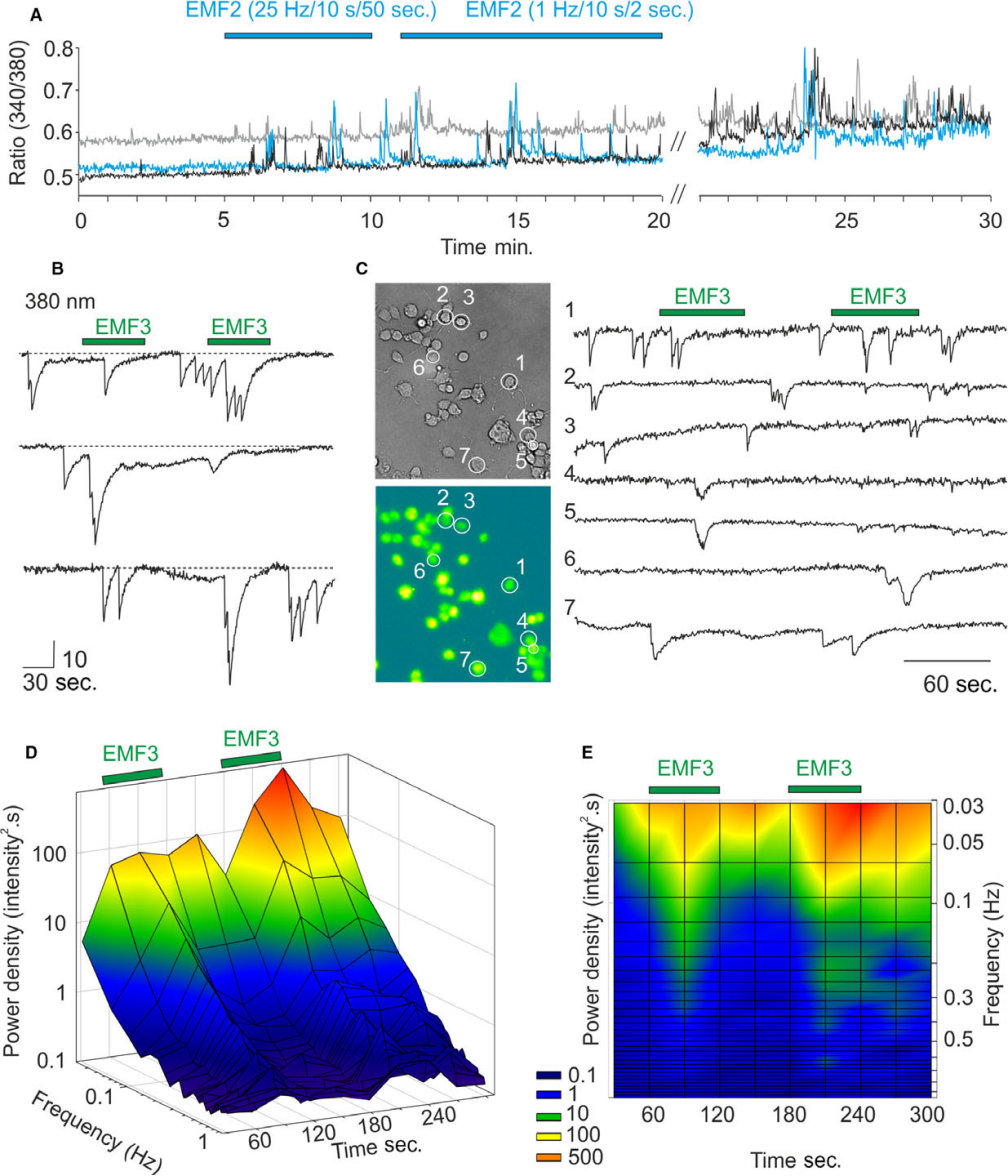
F11 cells exhibit spontaneous activity that is increased in response to LF‐EMF. (**A**) Spontaneous Ca^2+^ activity in three representative F11 cells exposed to EMF2 (with two different settings of parameters), measured as the relative calcium increase, expressed by the 340/380 fluorescence ratio. (**B**) Spontaneous Ca^2+^ activity in three representative F11 cells exposed to EMF3. The spontaneous activity could be monitored at a better time resolution using one excitation wavelength (380 nm) and the 500‐msec. sampling rate. The vertical scale is in arbitrary intensity units. (**C**) Recording from seven representative Fura‐2‐loaded cells exposed twice to 60 sec. to EMF3 (indicated by green bar above the records). (**D**) and (**E**) The spontaneous Ca^2+^ activity was analysed using Fast Fourier Transformation (using Von Hann windowing function available in Clampfit). Average power spectrum obtained from 154 cells reveals a clear and reversible increase in activity upon EMF3 stimulation.

We regularly observed spontaneous Ca^2+^ activity in a subset of F11 cells (Fig. 2A) that could be better monitored using one excitation wavelength (380 nm) and subsecond sampling rates (Fig. 2B). The occurrence of spontaneous activity depended on the extent of differentiation of F11 cells. The highest activity was observed in cells 4–8 days after passaging. The percentage of spontaneously active cells was 19 ± 3% (calculated from 2363 cells from 28 independent experiments conducted at eight different days). Although not obvious at first sight, the spontaneous activity seemed to be promoted by EMF2 and EMF3, but this effect was not apparently reversible and was seen only in some cells. In a small proportion of cells (21/894 in case of EMF3), switching off the magnetic field, on the contrary, increased the activity. To quantify these observations, we decided to apply EMF3 that had most pronounced effects on F11 responses (Fig. 2B and C), and the Fast Fourier Transformation that converts a signal to its representation in the frequency domain. The resulting average power spectra obtained from 154 cells exposed twice to ELF3 for 60 sec. with 60‐sec. interval revealed a clear increase in power spectral density within the frequency range of ~0.03–0.3 Hz (Fig. 2D and E).

### LF‐EMF affects crosstalk among F11 cells heterologously expressing human transient receptor potential TRPA1

**Figure 3:**
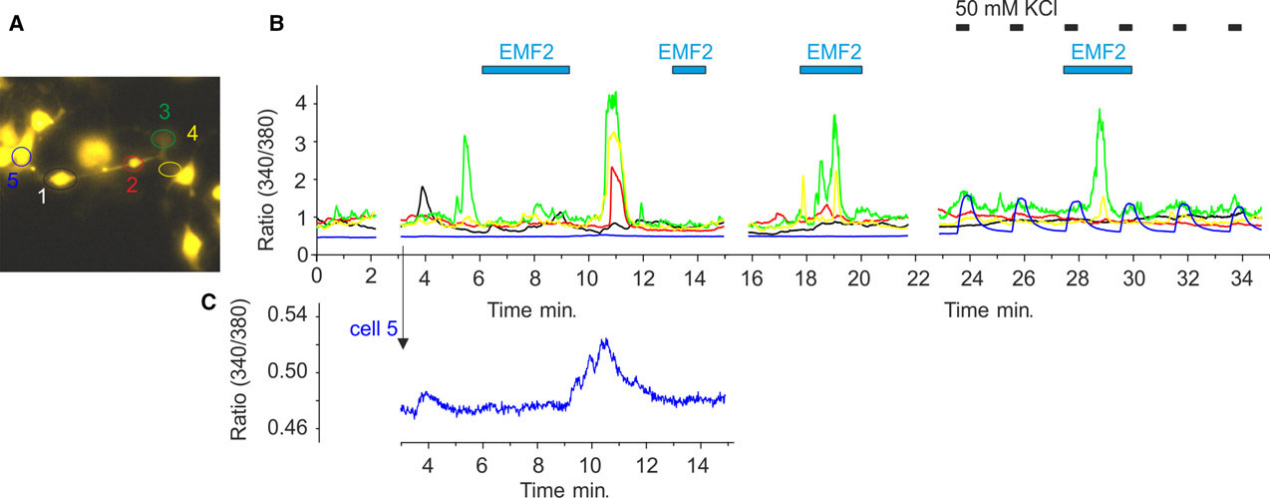
Crosstalk among interconnected F11 cells is affected by LF‐EMF. (**A**) F11 cells transfected with human TRPA1. (**B**) Time course of Fura‐2 ratiometric responses from cells shown in A. Cell 1 (black line) and cell 2 (red line) were GFP positive and, thus, most likely, expressed TRPA1. Ca^2+^ responses to EMF2 (intensity 79%, frequency 25 Hz, packet 10 sec., pause 50 sec.). The cell 5 (blue trace) which exhibited a lower basal level of intracellular Ca^2+^ was affected after switching off the EMF2 stimulator. This portion of the graph is enlarged below (**C**). The low activity of cell 5 could trigger the robust Ca^2+^ transients in cells 2, 3 and 4. The cell 5 responded to repeated application of 50 mM KCl (horizontal bars above the record). Similar effects were seen in three independent experiments.

The reported beneficial effects of high‐induction electromagnetic field on acute and persistent pain 11, 12, 13, 14, 15 prompted us to further explore whether LF‐EMF may be able to influence some of the known prototypical nociceptor‐specific ion channels. As an example, we have selected the transient receptor potential ankyrin subtype 1 (TRPA1) which is an excitatory ion channel expressed by nociceptive neurons of the dorsal root and trigeminal ganglia. There it mediates acute and chronic pain and plays an important role in initiation and maintenance of chronic inflammatory diseases and tissue injuries (e.g. Ref. 28, 29, 30, 31, 32, 33 and references therein). TRPA1 transcript is completely absent in F11 cells 19; therefore, these cells are well suitable for studying this channel under close‐to‐native conditions 34. We co‐expressed human TRPA1 with green fluorescent protein (GFP, as a marker) and measured Ca^2+^ responses to EMF2. As we previously described 34, TRPA1‐expressing cells exhibited increased basal levels of intracellular Ca^2+^ and we occasionally observed spontaneous calcium transients in a subset of cells (usually 1–2 per experiment). Figure 3 shows a representative recording obtained from one of three independent experiments. Among five interconnected cells, cell 1 (black line) and cell 2 (red line) were GFP positive and, thus, most likely, expressed TRPA1 channels. Prolonged application of EMF2 did not induce any response, but after discontinuation of the magnetic field, calcium transients occurred simultaneously in three of the cells. Six minutes later, EMF2 triggered activity in these cells, but it did not affect those cells that exhibited low basal Ca^2+^ levels. Such cells consistently responded to repeated application of 50 mM KCl (see cell 5 in Fig. 3B and C).

## Discussion

Together, our results demonstrate that repetitive electromagnetic stimulation has acute effects on calcium responses in model peripheral sensory neurons. We show that short‐term exposure of naïve F11 cells to LF‐EMF reduces calcium transients in response to bradykinin (Fig. 1) and demonstrate a potentiating effect of LF‐EMF on spontaneous activity of F11 cells under two different conditions (Figs. 2 and 3). The main result of this pilot study is the demonstration that sensory neuron‐derived cell line is a suitable and physiologically relevant tool for studying the mechanisms underlying the effects of LF‐EMF at the cellular and molecular level. The information gleaned from studies on the cellular processes that might be involved in such effects is scattered in the literature and does not permit yet to integrate the results into an explicit mechanism for several main reasons: (i) the cell types, (ii) parameters of electromagnetic field, (iii) the length of exposure, (iv) the pharmacological and biochemical tools and (v) a biased focus on only one aspect of cellular responsiveness were different in each study. The identification of a plausible mechanism that could be extrapolated in the therapeutic context can be still quite difficult due to the complexity of effects. We demonstrate that exposure to high‐induction EMF attenuates responses to 10 nM bradykinin. Under voltage‐clamp conditions, bradykinin reduced Na^+^ inward currents and increased the resting membrane current, indicating alterations in neuronal excitability. Bradykinin stimulates the hydrolysis of phosphatidylinositol 4,5‐bisphosphate. This membrane phospholipid is a necessary and general cofactor whose requirement is clearly established for many ion channels, receptors and transporters 23, 35, 36. Thus, to further explain the multiple mechanisms whereby LF‐EMF may affect the cellular signalling would require careful characterization of responses in a particular native cell type and the use of novel approaches to identify receptors and cellular pathways involved. Interestingly, Ambrosino *et al*. 22 previously reported that Ca^2+^ responses do not desensitize when BK is repeatedly applied on F11 cells at a concentration of 250 nM. The authors used three subsequent BK exposures, each lasting 30 sec. and separated by >15 min. This time interval was probably sufficient for full resensitization of BK receptors and their signalling pathways, in contrast to 3‐min. interval used in our application protocol. Whereas LF‐EMF seemed to affect the signalling pathways involved in the desensitization, the effects on resensitization cannot be assessed from our recordings. Given that the bradykinin B2 receptors exhibit two different apparent affinities in F11 cells (EC_50_s of 1.8 × 10^−9^ and 3.7 × 10^−12^ M) 20, the strong desensitization of Ca^2+^ responses observed in our experiments indicates that LF‐EMF may affect the higher affinity signalling pathway of the BK receptor.

We examined a possible contribution of TRPA1 in EMF‐induced responses (Fig. 3). This ion channel is considered to be mechanosensitive 37, 38, and we hypothesized that such a channel might be also physically prone to be modulated by high‐induction electromagnetic field. The overexpression of TRPA1 heightened the sensitivity of F11 cells to EMF, but this effect was rather due to increased basal levels of intracellular Ca^2+^. Additional studies, which are currently ongoing, are necessary to further elucidate whether and to what extent LF‐EMF may influence other mechanotransduction ion channels that modulate nociceptor excitability and/or contribute to action potential propagation in sensory neurons 39. Such ion channels are expected to be intrinsically capable of stimulus‐specific conformational changes, and their putative involvement in magnetosensation would take us one step closer to understanding how these processes might occur.

## Conflict of interest

The authors confirm that there is no conflict of interests.
